# HIF1A contributes to the survival of aneuploid and mosaic pre-implantation embryos

**DOI:** 10.1101/2023.09.04.556218

**Published:** 2024-07-19

**Authors:** Estefania Sanchez-Vasquez, Marianne E. Bronner, Magdalena Zernicka-Goetz

**Affiliations:** 1 Division of Biology 139-74, California Institute of Technology, Pasadena, CA 91125, USA.; 2 Department of Physiology, Development and Neuroscience, University of Cambridge, Cambridge, UK.

**Keywords:** Embryogenesis, Aneuploidy, Mosaic embryos, DNA repair, Hypoxia

## Abstract

Human fertility is suboptimal, partly due to error-prone divisions in early cleavage-stages that result in aneuploidy. Most human pre-implantation are mosaics of euploid and aneuploid cells, however, mosaic embryos with a low proportion of aneuploid cells have a similar likelihood of developing to term as fully euploid embryos. How embryos manage aneuploidy during development is poorly understood. This knowledge is crucial for improving fertility treatments and reducing developmental defects. To explore these mechanisms, we established a new mouse model of chromosome mosaicism to study the fate of aneuploid cells during pre-implantation development. We previously used the Mps1 inhibitor reversine to generate aneuploidy in embryos. Here, we found that treatment with the more specific Mps1 inhibitor AZ3146 induced chromosome segregation defects in pre-implantation embryos, similar to reversine. However, AZ3146-treated embryos showed a higher developmental potential than reversine-treated embryos. Unlike reversine-treated embryos, AZ3146-treated embryos exhibited transient upregulation of Hypoxia Inducible-Factor-1A (HIF1A) and lacked p53 upregulation. Pre-implantation embryos develop in a hypoxic environment *in vivo*, and hypoxia exposure *in vitro* reduced DNA damage in response to Mps1 inhibition and increased the proportion of euploid cells in the mosaic epiblast. Inhibiting HIF1A in mosaic embryos also decreased the proportion of aneuploid cells in mosaic embryos. Our work illuminates potential strategies to improve the developmental potential of mosaic embryos.

## INTRODUCTION

Humans exhibit suboptimal fertility relative to other mammals (Palmerola et al., 2022). Only ~30% of human conceptions progress to live birth (Capalbo et al., 2021). Intriguingly, the first zygotic cleavage-stage divisions in human embryos are error prone, generally resulting in aneuploidy, in which cells gain or lose chromosomes (Allais and FitzHarris, 2022; Currie et al., 2022; Palmerola et al., 2022; Pauerova et al., 2020; Vanneste et al., 2009). A high incidence of aneuploidy in the early cleavage divisions is thought to underlie low human fecundity and most developmental defects (Palmerola et al., 2022). Both *in vivo* and *in vitro* fertilization (IVF) often give rise to mosaic embryos of diploid and aneuploid cells. Although it is predicted that ~60% of pre-implantation IVF embryos exhibit diploid-aneuploid mosaicism (Capalbo et al., 2021), our understanding of the embryo’s ability to cope with such abnormalities is limited.

The incidence of aneuploidy declines at later stages of development (Shahbazi et al., 2020), but the mechanism underlying this decline is poorly understood. Human mosaic embryos can develop to term (Capalbo et al., 2021; Greco et al., 2015; Starostik et al., 2020). Specifically, implantation of low- and medium-grade mosaic embryos, defined as having 20–30% and 30–50% aneuploid extra-embryonic trophectoderm cells in a biopsy, has been reported to be as likely to result in the birth of healthy babies as implantation of fully euploid embryos (Capalbo et al., 2021). Given that some mosaic embryos are able to develop to term, it is important to uncover the mechanisms that confer their viability.

Mouse models of chromosome mosaicism enable analyses that are not allowed with human embryos for ethical reasons. Mouse and human pre-implantation development are very similar to each other: both undertake cleavage divisions, compaction, blastocyst cavity formation and hatching, albeit with slightly different timings in the two species (Mole et al., 2020; Zhu et al., 2021). During pre-implantation development, cells on the outside of the embryo form the extra-embryonic trophectoderm (TE) whereas cells on the inside form the pluripotent inner cell mass (ICM). Subsequently, cells of the ICM are segregated into the embryonic epiblast (EPI) and extra-embryonic primitive endoderm (PE). The TE will form the placenta, the PE will form the yolk sac and the EPI will form the fetus (Zhu and Zernicka-Goetz, 2020).

Recently, our group developed the first mouse model of chromosome mosaicism, based on the generation of aneuploidies induced by a drug reversine (Bolton et al., 2016; Singla et al., 2020). Reversine is a pan-Aurora kinase inhibitor that also antagonizes the A3 adenosine receptor and inhibits mitotic kinase monopolar spindle 1 (MPS1) (D’Alise et al., 2008; Santaguida et al., 2010). Our analysis revealed that reversine-treated mosaic embryos are aneuploid and overexpress p53 (Bolton et al., 2016). Importantly, reversine-treated cells in mosaic embryos are gradually eliminated from the EPI, particularly around the time of blastocyst implantation. As with human mosaic embryos (Capalbo et al., 2021), we found that mosaic embryos with at least 50% of euploid cells had a similar developmental potential as fully euploid embryos (Bolton et al., 2016; Singla et al., 2020).

Because reversine affects p53 and may compromise cellular fitness (D’Alise et al., 2008; Santaguida et al., 2010), we sought to generate a complementary aneuploid model. To this end, we used the more specific Mps1 inhibitor, AZ3146 (Hewitt et al., 2010). Although AZ3146 and reversine both interfere with the spindle assembly checkpoint (Hewitt et al., 2010), they bind to Mps1 in a different manner (Lan and Cleveland, 2010). In mouse embryos, AZ3146 treatment was shown to double the occurrence of micronuclei, a marker of chromosome segregation defects, but without negatively affecting cellular fitness (Vazquez-Diez et al., 2019). In the present study, we used AZ3146- and reversine-treated pre-implantation mouse embryos to dissect the mechanisms underlying aneuploid cell elimination and survival.

## RESULTS

### AZ3146 treatment induces chromosome segregation defects in pre-implantation mouse embryos.

**Figure 1|:**
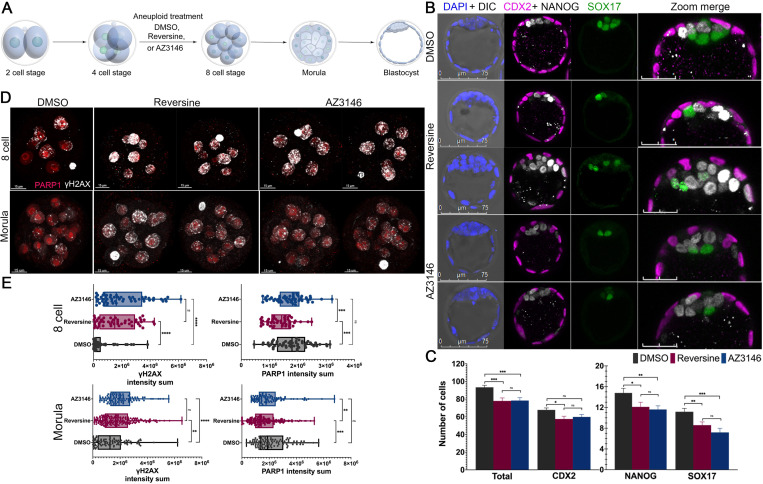
Lineage analysis of aneuploid embryos generated by selective Mps1 inhibitors AZ3146 and reversine. (A) Graphic representation of 4-cell embryos treated with DMSO (control) or Mps1 inhibitors reversine (0.5 μM) and AZ3146 (20 μM) to inactivate the spindle assembly checkpoint (SAC) and induce chromosome segregation errors. After washing, embryos were cultured to the mature blastocyst stage (E4.5) and analyzed for lineage specification. (B) Immunofluorescence imaging of well-known lineage markers CDX2 (TE), NANOG (EPI) and SOX17 (PE) reveals that overall embryonic morphology and cavitation is not affected by Mps1 inhibition. (C) Number of cells in each lineage was quantified to evaluate the effect of drug treatment on blastocyst development. Importantly, both reversine and AZ3146-treatments reduce the number of cells in the ICM, marked by NANOG (EPI) and SOX17 (PE). Whereas the TE, marked by CDX2, is reduced only in the reversine-treated embryos. (n=28 per treatment) (***P<0.0001, **P<0.01, *P<0.05, Mann–Whitney U-test, error bars represent s.e.m). (D) Analysis of DNA damage and DNA repair based on immunofluorescence against γH2A.X (phosphorylated form of H2A.X, white) and PARP1 (red), respectively. (E) Intensity analysis shows that reversine and AZ3146 increase DNA damage at the 8-cell stage through morula stage. Importantly, reversine appears to downregulate PARP1 expression at the 8-cell stage, which extends to morula stage embryos (***P<0.0001, **P<0.01, *P<0.05, t-test).

To generate distinct models of aneuploidy, we treated 4- to 8-cell stage mouse embryos with AZ3146 (20 μM) (Vazquez-Diez et al., 2019), as well as with reversine (0.5 μM) (Bolton et al., 2016) as a positive control, or DMSO (vehicle) as a negative control (Fig. 1A). We evaluated how the different Mps1 inhibitors affect chromosome segregation by detecting nuclei and kinetochores in 8-cell embryos and counted chromosomes *in situ* (Pauerova et al., 2020). Micronuclei were identified as small DAPI-stained chromosomes that were clearly distinct from the nuclei. We examined 32 DMSO-treated control embryos and observed only 22 cells with micronuclei out of a total of 256 cells (Fig. S1A). In contrast, we observed 82 cells with micronuclei in a total of 144 individual cells from 18 reversine-treated embryos, and 182 cells in a total of 304 individual cells from 38 AZ3146-treated embryos. Overall, a median of 12.5% of blastomeres had micronuclei in DMSO-treated 8-cell embryos, compared to 75% of blastomeres in the reversine-treated embryos and 62.5% of blastomeres in the AZ3146-treated embryos (Fig. S1B). We also detected non-dividing nuclei as rounded circles with distinct DAPI intensities in embryos treated with reversine and AZ3146). Specifically, we observed 28 non-dividing cells (19%) in reversine-treated embryos and 24 non-dividing cells (7.9%) in AZ3146-treated blastomeres, but none in DMSO-treated blastomeres (Fig. S1B). The increased frequencies of micronuclei and non-dividing cells in response to Mps1 inhibition are consistent with elevated aneuploidy and chromosomal instability (Daughtry and Chavez, 2016; Vazquez-Diez et al., 2016), as we showed previously for reversine-treated embryos (Bolton et al., 2016).

Given these distinct responses to reversine and AZ3146, we examined how these treatments impact the lineages in the blastocyst. To this end, we treated embryos with DMSO, reversine or AZ3146 from the 4- to 8-cell stage, and then washed and cultured them until the late blastocyst stage (E4.5). We assessed lineages by immunofluorescence (IF) to detect the TE marker CDX2, the EPI marker NANOG, and the PE marker SOX17. We found that all lineages segregated normally, and that blastocyst morphology was similar in all three conditions (Fig. 1B). We quantified the number of cells in the blastocysts and found that DMSO-treated controls had a median of 93 cells in total, whereas reversine- and AZ3146-treated embryos had a median of only 79 and 82 cells, respectively (***P<0.0001, **P<0.01, *P<0.05, Mann–Whitney U-test, n=28 per treatment) (Fig. 1C). DMSO-treated blastocysts contained a median of 15 EPI, 11 PE, and 67 TE cells, whereas reversine-treated blastocysts contained 12 EPI, 8 PE and 59 TE cells, and AZ3146-treated blastocysts had 11 EPI, 7 PE and 64 TE cells. These data suggest that reversine treatment compromises the development of all three lineages, as observed previously (Bolton et al., 2016), whereas AZ3146 mainly compromises ICM (EPI and PE) development.

To compare the developmental potential of blastocysts that had been treated with AZ3146 or reversine, we transferred them into opposite uterine horns of the same mouse and counted decidua, which reflect successful implantation, as well as viable embryos at E9.5. Although two (8%) reversine-treated blastocysts developed decidua, none gave rise to viable E9.5 embryos (Fig. S1C). In contrast, seven (30%) AZ3146-treated blastocysts developed decidua and five (21%) generated viable E9.5 embryos (Fig. S1D). Thus, AZ3146-treated embryos appear to have a higher developmental potential than reversine-treated embryos.

Considering that cellular fitness and aneuploid stress are related to DNA damage and DNA repair, we first sought to understand if these parameters were affected in our drug treatments. We used IF to analyze the levels of the DNA repair marker PARP1 and of the DNA damage marker, phosphorylated H2A.X (γH2A.X) at the 8-cell and morula stages (Fig. 1D). We observed elevated γH2A.X levels in reversine- and AZ3146-treated 8-cell embryos compared to controls (Fig. 1D), which eventually returned to normal in AZ3146-treated blastocysts, but not in reversine-treated blastocysts (Fig. S2A).

PARP1 was specifically enriched in the EPI lineage in normal blastocysts, as assessed by IF and re-analysis of published scRNA-seq data (Deng et al., 2014) (Fig. S2B). Intriguingly, PARP1 levels were reduced at the 8-cell and morula stages in reversine-treated embryos compared to controls and to AZ3146-treated embryos (Fig. 1 D-E). Moreover, late morula stage embryos treated with the PARP inhibitor Olaparib (10 μM, Hou et al., 2022; Prasad et al., 2017) developed into smaller blastocysts with only 78 total cells, reflecting a relatively low number of ICM cells, only 8 EPI and 4 PE cells (Fig. S2C-E). Treatment with reversine, but not AZ3146, further reduced the number of cells in the EPI lineage of Olaparib-treated embryos (Fig. S2E). Overall, these data suggest that PARP1 is required for proper development of the ICM, and that its reduced levels after reversine treatment may negatively impact development.

### Reversine and AZ3146 activate distinct stress response pathways in pre-implantation embryos.

Chromosome mis-segregation and aneuploidy are associated with different cellular stress pathways (Zhu et al., 2018). For instance, p53 is frequently activated following DNA damage, which can limit proliferation and trigger apoptosis (Abuetabh et al., 2022; Soto et al., 2017; Thompson and Compton, 2010). The p38 mitogen-activated protein kinase (MAPK) can also be activated following DNA damage (Thompson and Compton, 2010). In aneuploid cells, p38 promotes apoptosis by inhibiting the transcription factor Hif-1α (Simoes-Sousa et al., 2018), which otherwise promotes cell survival, proliferation, and metabolic changes in aneuploid cells and in response to hypoxia in different contexts (Hu et al., 2003; Simoes-Sousa et al., 2018).

To investigate how treatment with reversine or AZ3146 affect *p53* and *Hif1a* expression in mouse embryos, we performed RT-qPCR at the morula and blastocyst stages. We normalized to *Ppia* (Peptidylprolyl Isomerase A) mRNA, which is a stable reference gene in diploid and polyploid embryos (Gu et al., 2014).

**Figure 2 |:**
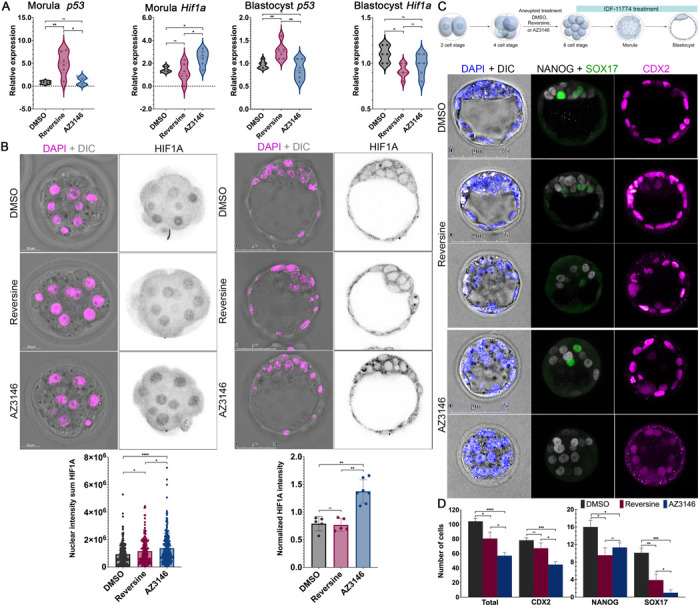
AZ3146-treated embryos elevate HIF1A activity to support formation of the TE and PE. A) qPCR analysis of *p53* and *Hif1a* mRNA expression at morula and blastocyst stages reveals that *p53* is upregulated in reversine-treated embryos and that *Hif1a* is upregulated in AZ3146-treated embryos at morula stages; 3 biological replicates and 2 technical replicates per experiment with each replicate having a minimum of 16 embryos (**P<0.01, *P<0.05, Welch’s t-test). B) Immunofluorescence against HIF1A (black) shows an increase in nuclear intensity in AZ3146-treated embryos at morula stages. At blastocyst stage, nuclear and cytoplasmic HIF1A are increased in AZ3146-treated embryos; normalization was based on DAPI staining (magenta). (C) Graphic representation of 4-cell embryos treated with DMSO and aneuploid drugs. Chemical downregulation of HIF1A was achieved by treatment with IDF-11774 immediately after wash of the aneuploid drugs. Immunofluorescence analysis of lineage specification in blastocyst cultured with the HIF1A inhibitor IDF-11774 using antibodies against CDX2 (TE), NANOG (EPI) and SOX17 (PE). Importantly, IDF-11774 appears to affect cavitation of some AZ3146-treated embryos. (D) Lineage analysis at the blastocyst stage shows that TE and PE specification are affected by IDF-11774 treatment. Number of cells in each lineage was quantified to evaluate the effect in blastocyst development. (***P<0.0001, **P<0.01, *P<0.05, Mann–Whitney U-test, error bars represent s.e.m).

Reversine-treated embryos displayed a significant increase in *p53* transcript levels at the morula (7-fold) and blastocyst (1.3-fold) stages (**P<0.01, *P<0.05, Mann–Whitney U-test) (Fig. 2A), as we showed previously (Singla et al., 2020). In addition, reversine-treated embryos showed reduced expression of *Hif1a* at the blastocyst stage, which would be consistent with p38 activation. In contrast, AZ3146-treated embryos did not show upregulation of *p53* at either the morula or blastocyst stage (Fig. 2A). Moreover, AZ3146-treated embryos showed a transient increase in *Hif1a* mRNA levels (3-fold) at the morula stage compared to DMSO- and reversine-treated embryos (Fig. 2A), which returned to normal levels at the blastocyst stage. HIF1A protein was present in DMSO- and reversine-treated embryos, and elevated at the morula and blastocyst stages in AZ3146-treated embryos (***P<0.0001, **P<0.01, *P<0.05, Mann–Whitney U-test) (Fig. 2B). HIF1A appeared to be mostly nuclear in morula, but mostly cytoplasmic in blastocysts, under all three conditions (Fig. 2B). Overall, these data suggest that treatment with reversine, but not AZ3146, induces multiple stress pathways in pre-implantation embryos. These differences may contribute to the increased developmental potential of embryos treated with AZ3146 versus reversine.

### HIF1A activity is required for proper blastocyst formation after Mps1 inhibition.

It was previously shown that Hif1α^−/−^ embryos undergo developmental arrest and lethality by E11 (Iyer et al., 1998). To assess the role of HIF1A in the embryo’s response to reversine and AZ3146, we used a pharmacological approach to inhibit its function. Briefly, we tested two different small molecules that have been shown to inhibit HIF1A activity, PX-478 (Lee and Kim, 2011; Zhao et al., 2015) and IDF-11774 (Ban et al., 2017). We treated control zygotes with DMSO, PX-478 (2 μM), or IDF-11774 (20 μM) until the blastocyst stage (Fig. S3A). Unlike PX-478, IDF-11774 treatment did not significantly affect the total number of cells in the TE or the whole blastocyst compared to the control (***P<0.0001, **P<0.01, *P<0.05, Mann–Whitney U-test) (Fig. S3B). Therefore, we used IDF-11774 to inhibit HIF1A in subsequent experiments.

Next, we treated embryos with DMSO, reversine, or AZ3146 from the 4- to 8-cell stage, washed them, and then inhibited HIF1A with IDF-11774 from the 8-cell to blastocyst stage (Fig. 2C). Inhibition of HIF1A did not abolish cavitation, but dramatically lowered the number of TE and especially PE cells in AZ3146-treated embryos compared to DMSO-treated controls (Fig. 2D) and compared to AZ3146-treated embryos not exposed to IDF-11884 (Fig. 1C). Inhibition of HIF1A also lowered the number of cells in reversine-treated embryos compared to controls, but the effect was smaller (Fig. 2D). Overall, our data suggest that elevated HIF1A activity from the 8-cell stage onwards is particularly important to promote the survival of aneuploid TE and PE cells after AZ3146 treatment.

Taken together, our data suggest that AZ3146 and reversine have distinct effects on the pre-implantation embryo. Compared to reversine-treated embryos, AZ3146-treated embryos appear to have increased developmental potential, lack upregulation of p53, and show transient upregulation of Hif1a. Moreover, AZ3146-treated embryos have a greater dependence on HIF1A activity to form the TE and PE.

### Hypoxia exposure attenuates DNA damage and blastomere defects in response to Mps1 inhibition.

**Figure 3 |:**
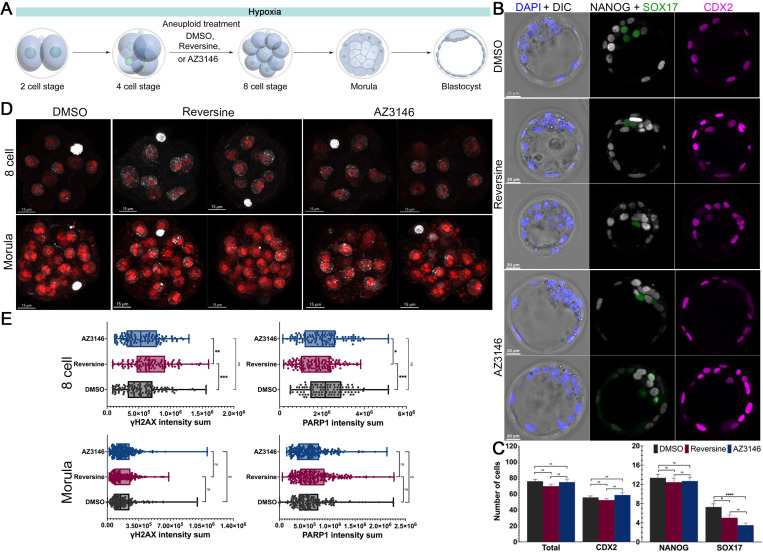
Hypoxia exposure reduces DNA damage and affects lineage proportions in the aneuploid blastocyst. (A) Graphic representation of the hypoxia experiments. 2-cell embryos were cultured until the blastocyst stage in hypoxia conditions (5% oxygen). As before, 4-cell stage embryos were treated with DMSO or Mps1 inhibitors reversine and AZ3146 until the 8-cell stage. After washing, embryos were cultured to the mature blastocyst stage (E4.5) and analyzed for lineage specification. (B) Immunofluorescence imaging of well-known lineage markers CDX2 (TE), NANOG (EPI) and SOX17 (PE) reveals that overall embryonic morphology and cavitation is not affected by Mps1 inhibition or hypoxia. (C) Lineage analysis at blastocyst stage. Number of cells in each lineage was used to evaluate the effects in blastocyst development. (*P<0.05, Mann–Whitney U-test, error bars represent s.e.m). (D) Immunofluorescence against PARP1 (red) and γH2A.X (white) in blastocyst after drug treatments. (E) Intensity analysis shows that, under hypoxia, DNA damage is only slightly increased at the 8-cell stage after exposure to reversine and AZ3146. PARP1 expression is altered only at the 8-cell stage in reversine-treated embryos (***P<0.0001, **P<0.01, *P<0.05, t-test).

Pre-implantation development occurs in a hypoxic environment, and many clinics culture human embryos under hypoxic conditions (Houghton, 2021). It was reported that physiologic oxygen concentrations can improve the yield and quality of mammalian blastocysts (Ciray et al., 2009; Nguyen et al., 2020) and increase the nuclear translocation of HIF1A in mouse blastocysts (Choi et al., 2021) To investigate how hypoxia affects the development of euploid and aneuploid mouse embryos, we cultured embryos from the 2-cell stage under hypoxic (5% O_2_/5% CO_2_) and normoxic (air, 20% O_2_/5% CO_2_; control) conditions until the blastocyst stage, treating them with DMSO, reversine, or AZ3146 from the 4–8 cell stage (Fig. 3A).

First, we examined lineage specification by quantifying the cell numbers in all three lineages at the blastocyst stage. We found that DMSO-treated blastocysts cultured under hypoxia had a median of 74 cells, representing 14 EPI cells, 6 PE cells and 54 TE cells (Fig. 3B-C). These data suggest that, under our conditions, the cell number in the pre-implantation mouse blastocyst is reduced when cultured in hypoxia compared to normoxia (Fig. 3C, 1C), particularly in the TE and PE. Notably, reversine and AZ3146 treatment did not further reduce cell numbers for embryos cultured under hypoxia, except for in the PE (Fig. 3C).

To investigate how hypoxia exposure affects DNA damage and repair in mouse embryos, we again performed IF for γH2A.X and PARP1 in 8-cell embryos and morula. We found that hypoxia exposure lowered the number of γH2A.X foci under all conditions, and minimized the differences between DMSO, reversine and AZ3146 treatments (Fig. 3D-E). In addition, PARP1 levels were also similar under all conditions, and relatively low in morula. Overall, these data suggest that hypoxia exposure lowers the accumulation of DNA damage under all conditions.

### Hypoxia increases the proportion of euploid cells in the epiblast of mosaic blastocysts.

Despite the high incidence of mosaicism in human pre-implantation embryos, the fate of aneuploid cells in mosaic embryos is incompletely understood. We previously used reversine to generate a mouse model of pre-implantation chromosome mosaicism and found that aneuploid (Reversine-treated) cells are eliminated in the EPI of mosaic embryos via apoptosis, starting from the mature blastocyst stage (Bolton et al., 2016). Whether this response reflects reversine treatment specifically, or aneuploidy more generally, is not known. Moreover, how hypoxia versus normoxia affects the outcomes is also not clear.

**Figure 4 |:**
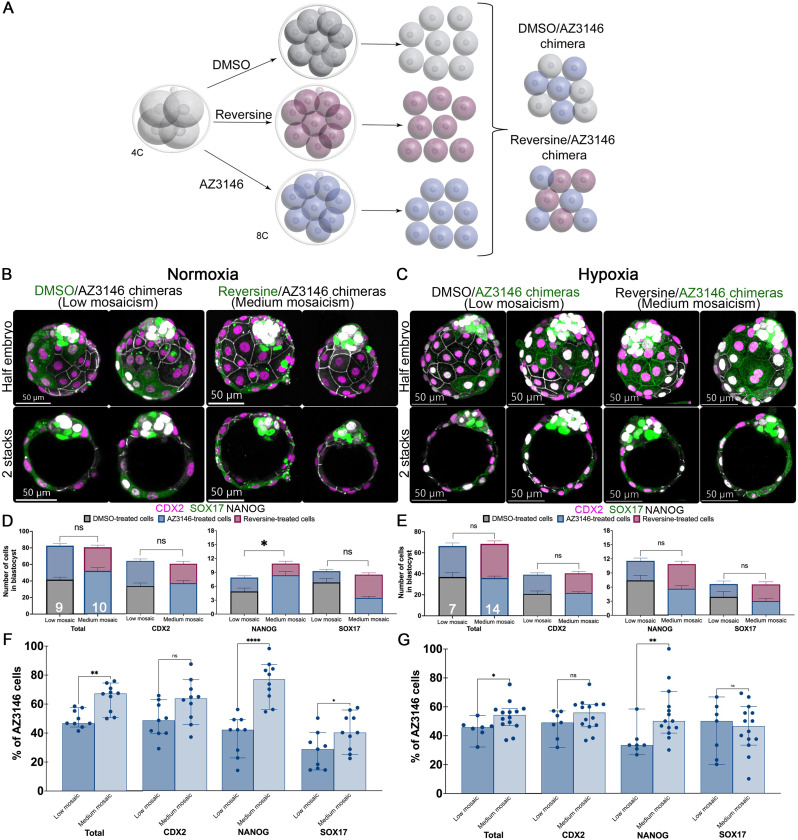
Hypoxia affects cell competition between diploid and aneuploid cells during pre-implantation development. (A) Graphic representation of the cell competition experiments. Embryos were treated at the 4-cell stage with DMSO or Mps1 inhibitors reversine and AZ3146. After washing, 8-cell stage embryos were disaggregated, and re-aggregated to form chimeras containing a 1:1 ratio of DMSO/AZ314-treated blastomeres and reversine/DMSO-treated blastomeres. Following aggregation, chimeras were cultured to the mature blastocyst stage (E4.5) and analyzed for lineage specification. For identification of the treatment, we use transgenics lines with membrane markers. Immunofluorescence for CDX2, NANOG, and SOX17 was performed to test lineage specification and allocation during (B) normoxia and (C) hypoxia. Lineage allocation quantification was based on the above markers. Importantly, D) in normoxia, both chimeras have the same number of cells in all the lineages except the EPI. In addition, AZ3146-treated blastomeres outcompete reversine-treated blastomeres in medium-grade mosaics in the TE and the EPI. E) Under hypoxia, both chimeras have similar number of cells in all the lineages. Yet, AZ3146-blastomeres do not outcompete reversine-treated blastomeres. Quantification of the contribution of AZ3146-treated blastomeres to the chimeras showed that, (F) under normoxia, compare with DMSO-treated cells, no preferential allocation of aneuploid cells occurs in the TE. In contrast, AZ3146-treated blastomeres increased their contribution when compare with reversine- treated blastomeres, but only significantly on the EPI and the PE. (G) During hypoxia, DMSO/AZ3146 chimeras did not change their behavior. Confirming a preferential allocation of diploid cells to the EPI. In reversine/AZ3146 chimeras, AZ3146-treated blastomeres contribute similarly to reversine-blastomeres to the TE and EPI but significantly increase contribution to the EPI. These results indicate that hypoxia favors the survival of reversine-induced aneuploid cells (Experiments were repeated in triplicate, ****P<0.00001, **P<0.01, *P<0.05, Mann–Whitney U-test, error bars represent s.e.m).

To address these questions, we created aggregation chimeras at the 8-cell stage that contained a 1:1 ratio of AZ3146-treated and control blastomeres (DMSO/AZ3146 chimeras), which is expected to reflect low-grade mosaicism, or of AZ3146-treated and reversine-treated blastomeres (reversine/AZ3146 chimeras), which is expected to reflect medium-grade mosaicism (Fig. 4A). We cultured these chimeric embryos in normoxia and hypoxia, and followed the fate of individual blastomeres by using transgenic mouse lines with the membrane markers mTmG (Muzumdar et al., 2007) and E-cadherin (Christodoulou et al., 2019). DMSO/AZ3146 and reversine/AZ3146 blastocysts displayed proper lineage allocation, embryo morphology and cavitation in both normoxia and hypoxia (Fig. 4B-C). In addition, DMSO/AZ3146 and reversine/AZ3146 embryos had comparable total cell numbers in their blastocysts, and blastocysts grown in hypoxia were again smaller than those grown in normoxia (Fig. 4D-E). Intriguingly, despite having fewer total cells, DMSO/AZ3146 blastocysts had more EPI cells when they were cultured in hypoxia compared to normoxia (Fig. 4D-E).

We quantified the proportion of AZ3146 cells in each lineage for each chimera. In DMSO/AZ3146-treated blastocysts, we found that 46.75% of the TE and 42.88% of the EPI originated from AZ3146-treated blastomeres, compared to only 28.57% of the PE (Fig. 4F). In reversine/AZ3146 chimeras, 63% of the TE and 78.4% of the EPI originated from AZ3146-treated cells, compared to only 40% of the PE (Fig. 4G). Thus, under normoxia, DMSO cells appear to outcompete AZ3146-treated cells, which in turn outcompete Reversine-treated cells in the TE and EPI. AZ3146-treated cells appeared to be at a competitive disadvantage in the PE in both contexts. Culturing DMSO/AZ3146 chimeras in hypoxia slightly increased the representation of AZ3146-treated blastomeres in the TE (49.06%) and PE (50%) but, strikingly, reduced their representation in the EPI (33.3%) (Fig. 4G). These altered frequencies reflect a higher number of DMSO-treated cells in the EPI and a lower number in the PE (Fig. 4E). Hypoxia exposure of reversine/AZ3146 embryos lowered the contribution of AZ3146-treated blastomeres to the TE (55.94%) and EPI (50%) but not the PE (46.43%) (Fig. 4G). We found that there was no correlation between the proportion of AZ3146-treated cells in the TE and the EPI in DMSO/AZ3146 and reversine/AZ3146 chimeras, under hypoxia or normoxia, and that hypoxia seemed to have a greater impact on the proportion of AZ3146 cells in reversine/AZ3146 chimeras (Fig. S4). Overall, these data suggest that hypoxia has lineage-specific effects on competitions between aneuploid and euploid cells and increases the contribution of euploid cells to the EPI.

Blastomeres in the 4-cell stage embryo display a lineage bias (Goolam et al., 2016). We considered that mosaicism generated before the 4-cell stage might influence lineage allocation. To test this possibility, we treated zygotes with reversine or AZ3146 during the first cell division. Importantly, reversine treatment at the 2-cell stage seems to strongly affect the morphology of the blastocysts (Fig. S5A). Consistent with this change in morphology, reversine-treatment reduced the number of cells in all lineages, particularly in the TE and PE (Fig. S5B). In contrast, AZ3146 treatment did not affect morphology or cell number in any of the lineages (Fig. S5A-B) (n>27 per treatment, ***P<0.0001, **P<0.01, *P<0.05, Mann–Whitney U-test). To evaluate how early generation of aneuploidies in the embryos affect cell competition, we generated DMSO/AZ3146, reversine/DMSO, and AZ3146/reversine aggregation chimeras at the 2-cell stage and cultured them under normoxia until the blastocyst stage (Fig. S5C). We found that all three 2-cell stage aggregation chimeras developed into blastocysts with a normal morphology and cavitation (Fig. S5D). Interestingly, the reversine-treated cells were extruded from the blastocyst in 54% of the reversine/DMSO chimeras and in 33% of the AZ3146/reversine chimeras, whereas no cell extrusion was observed in the DMSO/AZ3146 chimeras. Quantification of the proportion of AZ3146-treated cells in 2-cell stage derived chimeras showed similar results as 8-cell stage derived chimeras (Fig. S5E). In DMSO/AZ3146-treated blastocysts, we found that 49.28% of the TE, 39% of the EPI and 33.33% of the PE originated from AZ3146-treated blastomeres (Fig. S5E). In reversine/AZ3146 chimeras, 68.24% of the TE, 68.83% of the EPI, and 66.67% of the PE originated from AZ3146-treated cells (Fig. S5E). In DMSO/reversine chimeras, 22.27% of the TE, 26.98% of the PE, and 31.7% of the EPI originated from reversine-treated cells. Overall, these results suggest that aneuploidy generated at different stages similarly affect the proportion of aneuploid cells in the blastocysts.

### HIF1A inhibition reduces the frequency of aneuploid cells in DMSO/AZ3146 mosaic embryos.

**Figure 5|:**
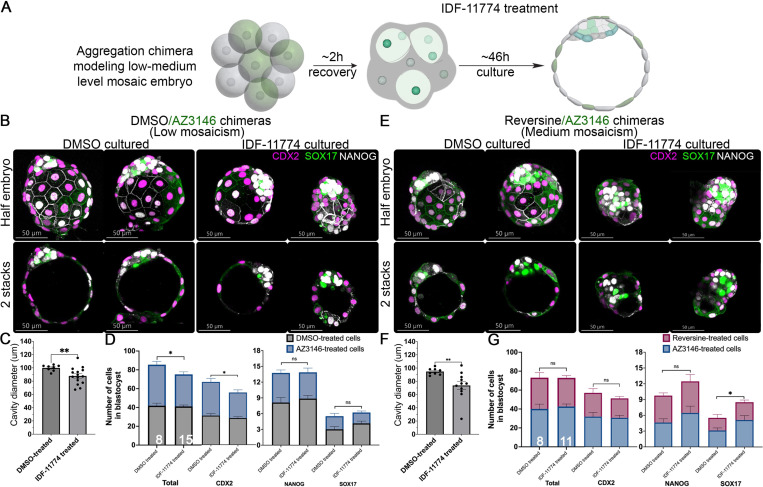
HIF1A inhibition increases the proportion of euploid cells in mosaic embryos. (A) Schematic of HIF1A inhibition by IDF-11774 in 8-cell stage aggregation chimeras cultured in hypoxia. Immunofluorescence for CDX2, NANOG, and SOX17 was performed to test lineage specification and allocation. (B) HIF1A inhibition in low-grade mosaicism does not affect overall morphology but affects (C) cavitation. In contrast, (E) HIF1A inhibition in medium-grade mosaicism affects morphology, and (F) cavitation of the embryos. Lineage allocation quantification after IDF-1174 reveals a significant reduction of total cell number (D) in low-grade mosaicism as well as a reduction in cell number in the TE but not in the EPI and PE. In contrast, total cell number is not affected (G) in medium-grade mosaicism. However, IDF11-774 treatment appears to increase the cell number of the PE. These results show that HIF1A inhibition in hypoxic conditions differentially affect each type of mosaic embryos. Experiments were repeated in triplicate, **P<0.005, *P<0.05, Mann–Whitney U-test, error bars represent s.e.m.

Our data suggest that hypoxia affects aneuploid-euploid cell competition in mosaic embryos (Fig. 4). To further assess the effect of HIF1A and, considering most human embryos are culture under hypoxic conditions, we decided to evaluate the effect of inhibiting HIF1A in mosaic embryos cultured in hypoxic conditions. We assembled DMSO/AZ3146 and AZ3146/reversine 8-cell stage chimeras, treated them with the HIF1A inhibitor IDF-11774 (Fig. 5A, S6A-B), and then assessed lineage allocation in blastocysts. Treatment of DMSO/AZ3146 chimeras with IDF11774 reduced the cavitation diameter and diminished the contribution of AZ3146-treated cells to all three lineages, and DMSO-treated cells compensated for this loss in the PE and EPI (Fig. 5B-D, Fig. S6C). Treating AZ3146/reversine chimeras with IDF11774 compromised blastocyst morphology (Fig. 5E-F) and slightly increased the number of PE and EPI cells (Fig. 5G), without altering the frequency of AZ3146-treated cells (Fig. S6D). Taken together, our data suggest that HIF1A promotes the survival of AZ3146-treated cells in DMSO/AZ3146 chimeras, and suggest that inhibiting HIF1A could increase the proportion of karyotypically normal cells in mosaic embryos.

## DISCUSSION

Aneuploidy is a common event during pre-implantation development (Allais and FitzHarris, 2022; Currie et al., 2022; Palmerola et al., 2022). Here, we describe different strategies to generate aneuploidy in the early embryo. Our results suggest that: 1) the Mps1 inhibitor, AZ3146, generates aneuploid embryos with enhanced DNA repair and reduced stress responses when compared to reversine-treated embryos. 2) Lineage specific responses to aneuploidy are present during blastocyst development, as we showed previously (Bolton et al., 2016; Singla et al., 2020). 3) HIF1A contributes to the survival of TE and PE cells in AZ3146-treated embryos whereas PARP1 is particularly required in EPI cells in both reversine and AZ3146-treated embryos. 4) Hypoxia enhances DNA repair and affects aneuploid-euploid cell competition in mosaic embryos.

Reversine-treated mouse embryos upregulate the senescent markers p53 and p21 (Singla et al., 2020) and fail to give rise to viable embryos (Bolton et al., 2016). Our results further indicate that reversine downregulates PARP1, as previously observed in glioma (Hirakata et al., 2021). Thus, reversine treatment may compromise embryo development due to a combined increase in DNA damage and decrease in DNA repair. In contrast, embryos treated with AZ3146 do not reduce PARP1 levels and have elevated HIF1A, which appears to improve survival of TE and PE cells and developmental potential.

DNA repair pathways are enhanced under hypoxic environments in somatic cells (Marti et al., 2021; Nakamura et al., 2022; Pietrzak et al., 2018). Similarly, our data show that aneuploid embryos cultured in hypoxia had lower levels of DNA damage at the morula stage, in agreement with previous studies reporting lower levels of γH2A.X in mouse blastocysts recovered from the uterus and in blastocysts cultured in hypoxia compared to blastocysts cultured in normoxia (Houghton, 2021; Meuter et al., 2014). Interestingly, recent studies suggest that hypoxia favors HIF1A’s association with PARP1 in cancer cells, increasing DNA repair (Marti et al., 2021; Nakamura et al., 2022; Pietrzak et al., 2018). In the future, a deeper understanding of the regulation of DNA repair in pre-implantation stages would allow us to understand the molecular response of embryos to aneuploid stress.

Confined placental mosaicism is commonly observed in human pregnancies, and refers to instances where the placenta is aneuploid but the fetus is euploid (McCoy, 2017). We previously found that aneuploid TE cells have an increased cell cycle length and senescence, whereas aneuploid ICM cells have an increased frequency of apoptosis, in reversine-treated embryos. Therefore abnormal but viable TE cells could lead to confined placental mosaicism. Our results further suggest that aneuploid cells contributed mostly to the TE, followed by the EPI, are especially depleted from the PE in AZ3146-treated embryos under normoxia. Intriguingly, under hypoxia aneuploid cells were depleted from the EPI and increase in the PE. Overall our data suggest that hypoxia may affect cell competition in a lineage-specific manner in the pre-implantation embryo, supporting an increased representation of euploid cells in the EPI relative to the PE and TE.

Finally, our results confirm that the proportion of aneuploid cells in the EPI and TE do not correlate, consistent with aneuploid survival in the TE but elimination from the ICM. These results are not surprising considering the low aneuploidy concordance rates between TE and ICM in human mosaic aneuploid embryos (30–40%) (Dahdouh and Garcia-Velasco, 2021).

Since low- and medium- grade mosaic human embryos have similar developmental potential as fully euploid embryos (Capalbo et al., 2021; Greco et al., 2015), understanding the molecular pathways involved in the responses to aneuploidy may have translational implications. Given that the extent and proportion of aneuploid cells in mosaic embryos impact survival (Capalbo et al., 2021), we propose that a reduction in aneuploid cells in the blastocyst will favor a successful pregnancy. Here, we downregulated HIF1A as a strategy for reducing aneuploid cells number and found that treatment with IDF-11774 increased the proportion of euploid cells in mosaic embryos. In sum, our study reveals distinct environmental factors that affect the extent and proportion of aneuploid cells within mosaic embryos.

## MATERIALS AND METHODS

### Pre-implantation embryo culture

Mice were maintained according to national and international guidelines. Four- to six-week-old B6SJLF1/J female mice were injected with 7.5IU PMSG followed by 7.5 IU hCG 48 h later, to induce superovulation. The females were then mated with B6CBAF1/J males or were indicated, with E-cadherin GFP or mTmG transgenic males. Pre-implantation mouse embryos were recovered 24 h and 40 h after the hCG injection to obtain zygotes and 2-cell stage embryos, respectively in M2 medium (Sigma, M7167). We incubate the embryos in KSOM until 4-cell stage for the aneuploid treatment, around 53 h post hCG. When recovering zygotes, cumulus cells were removed with 0.3% hyaluronidase (Sigma, H4272) in M2. Embryos then were cultured in regular KSOM (Sigma. MR-106) at 37°C under 5% CO_2_/air (Normoxia) or with pre-mixed 5% CO_2_/ 5% O_2_ balance with nitrogen, biologic atmosphere batch (Airgas #Z03NI9022000033)(Hypoxia). B6SJLF1/J females and B6CBAF1/J males were obtained from JAX laboratories. Females were received at 3–4 weeks of age and were maintained in the CALTECH animal facility, where they were housed with 5 same-sex littermates on a 12 h light/12 h dark cycle with food and water *ad libitum*. The temperature in the facility was controlled and maintained at 21 °C. All experimental procedures involving the use of live animals, or their tissues were performed in accordance with the NIH guidelines and approved by the Institutional Animal Care and Use Committee (IACUC) and the Institutional Biosafety Committee at the California Institute of Technology (CALTECH). Reversine (Sigma, R3904), AZ3146 (Sigma, SML1427), PX-478 (Selleckchem, S7612), IDF-11774 (Selleckchem, S8771) and olaparib (Selleckchem, S1060) were dissolved in DMSO (Sigma, D2650) before use to specific concentration. They were respectively used at following final concentrations: 0.5 μM, 20 μM, 2 μM, 20 μM and 10 μM. Control embryos were incubated in the equivalent DMSO concentration. Drugs were dissolved in regular KSOM to the concentration of use. DMSO concentration in media should never pass 0.4%.

### Immunofluorescence

Embryos were fixed in 4% PFA (Thermo Scientific, AA47340) for 20 min at room temperature (RT). Following by three washes with 0.1% Tween-20 (Sigma, P1379) dissolved in PBS (PBST). The embryos were then permeabilized with 0.3%, Triton X-100 (Sigma, X100) in PBS. Washes were then performed three times in PBST before embryos were transferred to blocking solution (3% BSA in PBS) for at least 3h at RT. Incubation with primary antibodies was performed in blocking solution overnight at 4°C. Next day, washes were performed three times in PBST before incubation with Alexa Fluor secondary antibodies (Thermo Fisher Scientific, 1:500) in blocking solution for 2 h. Washes were performed three times before incubation with DAPI (Thermo Fisher Scientific) for 5 min. Washes after DAPI were perform two times in PBST before the final incubation in M2. Embryos were then mounted in M2 micro-drops on 35 mm glass bottom dish (MATTEK, P35G-1.5–14-C). Confocal imaging was carried out using Leica SP8, 40x objective, 1 μM Z-step. Image files were viewed and analyzed using ImageJ and IMARIS 9.9 software.

#### Primary antibodies used:

mouse anti-CDX2 (Biogenex, 1:500), rabbit anti-NANOG (1:500; Abcam), goat anti-SOX17 (1:300; R&D Systems), rabbit anti-HIF1A (1:300, Novusbio), mouse anti-PARP1 (1:500, Proteintech) and rabbit anti- Phospho- Histone H2AX (1:500, R&D Systems).

### *In situ* chromosome counting

For determination of aneuploidy, treated 8-cell stage embryos were synchronized in metaphase by 10h treatment with 0.03 μg/mL colcemid (Cayman, 15364) diluted in KSOM. Following by 1h treatment with 10 μM Mg132 (Selleckchem, S2619) in KSOM. Finally, 1 h treatment with 5 μM dimethylenastron (MedChemExpress, HY-19944) and 10 μM Mg132 in KSOM. Synchronized embryos were then fix in 2% PFA for 20 min, permeabilized with PBST for 15 min and blocked 3h at RT before incubation with human anti-centromere protein antibody (1:300, Antibodiesinc 15–234) overnight at 4°C. Next day, three washes with PBST were performed before 2 h incubation with goat anti-Human secondary antibody, Alexa Fluor 647 (1:400, Invitrogen A-21445) in blocking solution. Washes were performed three times before 20 min incubation with DAPI (1:500) and Alexa Fluor 488 Phalloidin (1:300, Invitrogen A12379). Washes then were performed three times before clearing overnight with AF1 plus (Citifluor, AF1/DAPI-15). Embryos were then mounted in AF1 plus micro-drops on 35 mm glass bottom. Confocal imaging was carried out using Leica SP8, 60x objective, 0.5 μM Z-step. Image files were viewed and analyzed using ImageJ and IMARIS 9.9 software.

### Embryo transfers and post implantation recovery and biopsy

Embryo transfer were performed as described previously(Bermejo-Alvarez et al., 2014). CD1 females and vasectomizes CD1 males were obtained from Charles River. Reversine and AZ3146-treated blastocyst were transfer into 2.5-days pseudopregnant CD1 females. Around 16 AZ3146-treated embryos were transfer in the right uterine horn, whereas around 16 reversine-treated embryos were transfer in the left uterine horn of the same female as control. To evaluate implantation and embryo survival potency, uterine horns were recovered 5 days after surgery. Deciduas were dissected and post implantation embryos at stage E9.5 were recovered into PBST on ice. Embryos were fixed in 4% PFA at RT for 1 h, followed by washes through PBST before imaging. Images were taken using an Olympus LS stereo microscope with a 10x objective. Uterine transfers were performed in accordance with the NIH guidelines and approved by the Institutional Animal Care and Use Committee (IACUC) and the Institutional Biosafety Committee at the California Institute of Technology (CALTECH).

### qRT-PCR

Around 14 to 17 morulas and blastocysts were collected for quantitative reverse transcriptase polymerase chain reaction (qRT-PCR). Total RNA from morulas was obtained using the NucleoSpin RNA Plus XS kit (Takara, 740990.10). Whereas total RNA from blastocyst was extracted using the Arcturus PicoPure RNA Isolation Kit (Thermo Fisher, KIT0204). qRT-PCR was performed using the *Power* SYBR Green RNA-to-CT 1-Step Kit (Applied Biosystems, 4389986) in a StepOne Plus Real-time PCR machine (Applied Biosystems). The following program was used: 30 min 48 °C (reverse-transcription) followed by 10 min 95 °C followed by 45 cycles of 15 s 95 °C (denaturing) and 1 min 60 °C (annealing and extension). The ddCT method was used to determine relative levels of mRNA expression, with Ppia as an endogenous control(Gu et al., ). Primers were obtained from IDT.

**Table:** 

Name	Sequence
p53_Fw	GTCACAGCACATGACGGAGG
p53_Rv	TCTTCCAGATGCTCGGGATAC
Hif1a_Fw	CCTGCACTGAATCAAGAGGTTGC
Hif1a_Rv	CCATCAGAAGGACTTGCTGGCT
Ppia_Fw	GAGCTCTGAGCACTGGAGAGA
Ppia_Rv	CCACCCTGGCACATGAAT

### Generation of chimeric embryos

Chimeric embryos were generated following a previous publish protocol(Eakin and Hadjantonakis, 2006). Briefly, wild-type, E-cadherin GFP or mTmG 8-cell stage embryos were transfer to micro-drops of M2 after aneuploid treatment. Zona pellucida was removed by treatment with acidic Tyrode’s solution (Sigma, T1788). The embryos were then incubated in Ca2+/Mg2+-free M2 (made in house) for 5 min and then disaggregated into individual blastomeres by gentle mouth pipetting. Low-grade mosaic chimeras were form by aggregation of four and four (1:1) of DMSO/reversine-treated blastomeres with AZ3146-treated blastomeres, respectively in KSOM. Medium-grade mosaic chimeras were form by aggregation of three DMSO-treated blastomeres, three AZ3146-treated blastomeres and two reversine-treaded blastomeres in KSOM. Culture of the chimeras were performed under normoxic and hypoxia condition in KSOM for 48 h to reach blastocyst stage.

### Statistical analysis

The statistical tests used are indicated in the corresponding figure legends. Calculations were carried out in Microsoft Excel and data analysis and visualization in Prism 9 software. All graphs show mean values, error bars: s.e.m.

## Supplementary Material

Supplement 1
