# Supplementary material for: HIF1A contributes to the survival of aneuploid and mosaic pre-implantation embryos
Source: bioRxiv. 2024 Jul 19:2023.09.04.556218. Preprint. [Version 2] doi: 10.1101/2023.09.04.556218 (PMC11275769; doi:10.1101/2023.09.04.556218)

bioRxiv preprint doi: https://doi.org/10.1101/2023.09.04.556218; this version posted July 19, 2024. The copyright holder for this preprint (which was not certified by peer review) is the author/funder, who has granted bioRxiv a license to display the preprint in perpetuity. It is made available under aCC-BY 4.0 International license.

Figure S.1| **AZ3146-treated embryos are aneuploid and can develop to post-implantation stages.**

bioRxiv preprint doi: https://doi.org/10.1101/2023.09.04.556218; this version posted July 19, 2024. The copyright holder for this preprint (which was not certified by peer review) is the author/funder, who has granted bioRxiv a license to display the preprint in perpetuity. It is made available under aCC-BY 4.0 International license.

(A) *In situ* chromosome counting of 8-cell stage embryos after drug treatments. Representative images of individual embryos in the different treatments. DNA (white) is visualized by DAPI staining and kinetochores (red) by CREST antibody. PB (polar body), MN (micronuclei). Each chromosome presents 2 CREST dots. The large number is the cell ID and the smaller number indicates the number of chromosomes, “n”. Diploid blastomeres present 40 pairs of CREST dots. Phalloidin (cyan) was used for cell segmentation. (B) Quantification of the incidence of aneuploid events, designated as those with more than 2 chromosomes (MN>2chr), less than 2 chromosomes (MN<2chr), without kinetochores (MN-CREST) and non-dividing nucleus (ND cell). We observed an increase in MN following reversine and AZ3146 treatment. Importantly, reversine-treated cells have a higher number of ND cells and MN-CREST staining. (n=128 cell DMSO treatment, n=152 cells AZ3146 treatment, n=72 cells reversine treatment, ****P<0.00001, **P<0.01, *P<0.05, Mann–Whitney U-test, error bars represent s.e.m). (C) Embryo transfer experiments show higher implantation events (% decidua formation) in AZ3146-treated embryos. The left uterine horn was used for transfer of reversine-treated blastocyst and the right uterine horn for AZ3146-treated blastocyst. (D) Only AZ3146-treated blastocyst can develop post-gastrulation E9.5 fetuses (% embryo formation).

Figure S.2| **PARP1 is required for ICM development**.
(A) Immunofluorescence against PARP1 (red) and γH2A.X (white) in blastocyst after drug treatments. Notice accumulation of DNA damage in reversine-treated blastocyst. (B) Immunofluorescence against PARP1 (red), NANOG (white, epiblast) and CDX2 (green, trophectoderm). Analysis of PARP1 intensity shows an increase in the EPI lineage. To confirm these observation, scRNA-seq (Deng et. al 2014) shows enrichment of *Parp1* mRNA specifically in the EPI. (C) Graphic representation of 4-cell embryos treated with DMSO and aneuploid drugs. Chemical downregulation of PARP1 was achieved by treatment with Olaparib from morula to blastocyst stage. (D) Effects of the inhibition of PARP1 function were assessed by immunofluorescence against CDX2 (TE), NANOG (EPI) and SOX17 (PE) in the different treatments. Importantly, Olaparib treatment does not affect blastocyst morphology. (E) Lineage analysis at blastocyst stage shows a specific effect on the survival of epiblast and PE cells in reversine-treated embryos. Ratio of cells in each lineage in the blastocyst is normalized based on DMSO treatment. (***P<0.0001, **P<0.01, *P<0.05, Mann–Whitney U-test, error bars represent s.e.m).

Figure S.3| **Pharmacological inhibitors of HIF1A have distinct effects on mouse pre-implantation embryos.** (A) Chemical inhibition of HIF1A from zygotes to blastocyst stage with PX-478 or IDF-11774. Immunofluorescence against CDX2 (TE) and DAPI (magenta) was used to assess blastocyst development. Importantly, IDF-11774 does not compromise pre-implantation development, whereas PX-478 compromised the survival and overall morphology of the blastocyst. (B) Analysis of the number of trophectoderm cells (CDX2) and total number (DAPI) shows a deleterious effect of PX-478 during blastocyst development (***P<0.0001, **P<0.01, *P<0.05, Mann–Whitney U-test, error bars represent s.e.m).

bioRxiv preprint doi: https://doi.org/10.1101/2023.09.04.556218; this version posted July 19, 2024. The copyright holder for this preprint (which was not certified by peer review) is the author/funder, who has granted bioRxiv a license to display the preprint in perpetuity. It is made available under aCC-BY 4.0 International license.

Figure S.4| **Correlation plot of AZ3146-treated cells in the epiblast and trophectoderm.** Scatter plot of AZ3146-treated cells in epiblast and trophectoderm in DMSO/AZ3146 mosaic blastocysts.

Figure S.5| **Aneuploidies generated at different stages have similar consequences on post-implantation development *in vitro*.** (A) Immunofluorescence for CDX2, NANOG, and SOX17 was performed in blastocysts after zygote treatment with Mps1 inhibitors. Importantly, reversine treatment at zygote stages affects morphology of blastocyst. (B) Lineage analysis of blastocyst shows no effect in AZ3146-treated embryos. Whereas reversine treatment reduces the cell number to almost half. Ratio of cells in each lineage in the blastocyst is normalized based on DMSO treatments. (n>27 per treatment) (***P<0.0001, **P<0.01, *P<0.05, Mann–Whitney U-test, error bars represent s.e.m). (C) Aggregation chimeras at 2-cell stage were created by using transgenics lines with membrane markers: mTmG (green) and E-cadherin (white). Subsequently, (D) immunofluorescence for CDX2, NANOG, and SOX17 was performed to quantify lineage allocation. Our results show no effect on embryo morphology in our chimeras. However, reversine/DMSO (D/R) chimeras seems to have increase events of cell extrusion and reversine/AZ3146 chimeras are smaller in number of cells compare with DMSO/AZ3146 chimeras. (E) Data show the proportion of AZ3146-treated cells in DMSO/AZ3146 mosaic blastocysts (grey) and in reversine/AZ3146 mosaic blastocysts (blue), as well as the proportion of reversine-treated cells in DMSO/reversine mosaic blastocysts (red).

Figure S.6| **IDF-11774-mediated inhibition of HIF1A increases the proportion of euploid cells in mosaic embryos.** Brightfield images of low- and medium-grade chimeras grown in (A) DMSO and (B) IDF-11774. Quantification of the contribution of AZ3146-treated blastomeres to the chimeras showed that, (C) in DMSO/AZ3146 mosaics, IDF-11774 treatment reduces the proportion of AZ3146-treated blastomeres, whereas (D) in reversine/AZ3146 mosaics, IDF-11774 treatment slightly increases the proportion of AZ3146-treated blastomeres. These results may indicate that HIF1A inhibition impairs the less fit population in chimeric embryos.

bioRxiv preprint doi: https://doi.org/10.1101/2023.09.04.556218; this version posted July 19, 2024. The copyright holder for this preprint (which was not certified by peer review) is the author/funder, who has granted bioRxiv a license to display the preprint in perpetuity. It is made available under aCC-BY 4.0 International license.

Figure S. 1

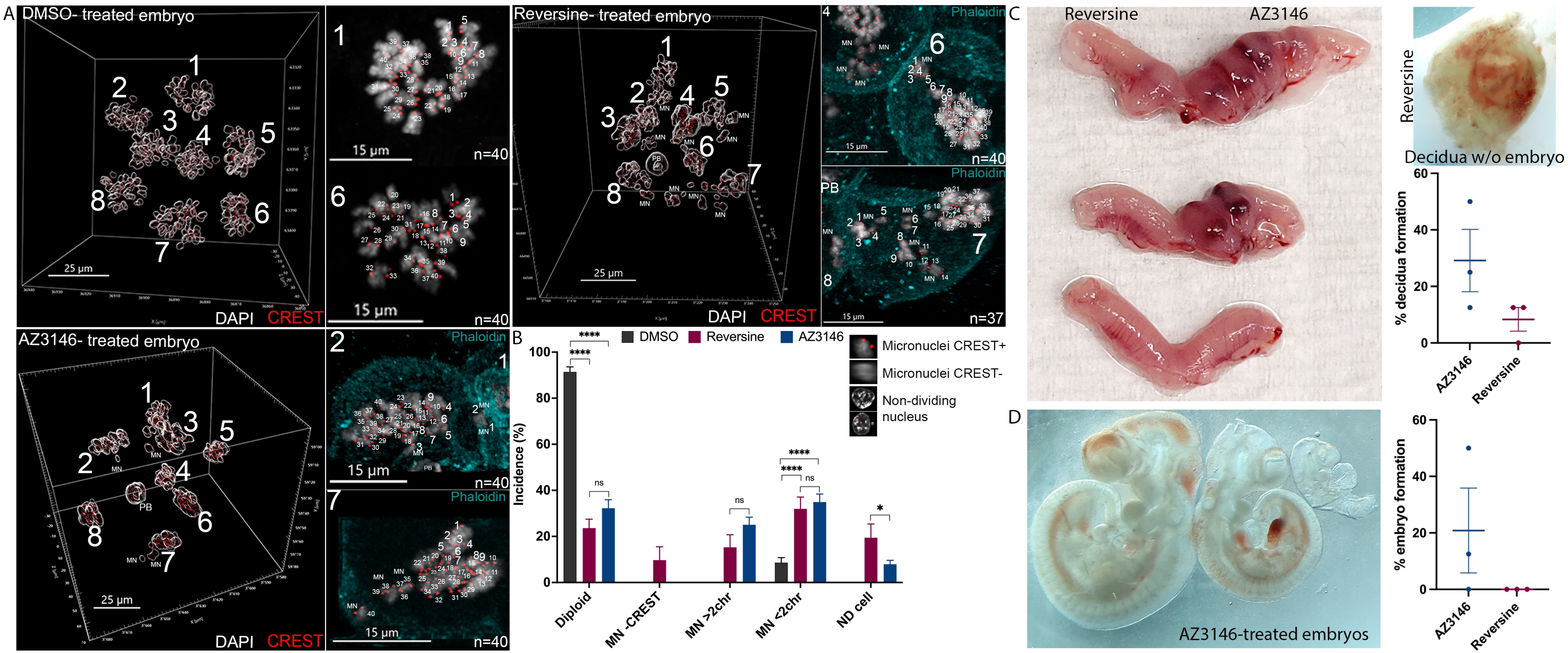

bioRxiv preprint doi: https://doi.org/10.1101/2023.09.04.556218; this version posted July 19, 2024. The copyright holder for this preprint (which was not certified by peer review) is the author/funder, who has granted bioRxiv a license to display the preprint in perpetuity. It is made available under aCC-BY 4.0 International license.

Figure S.2

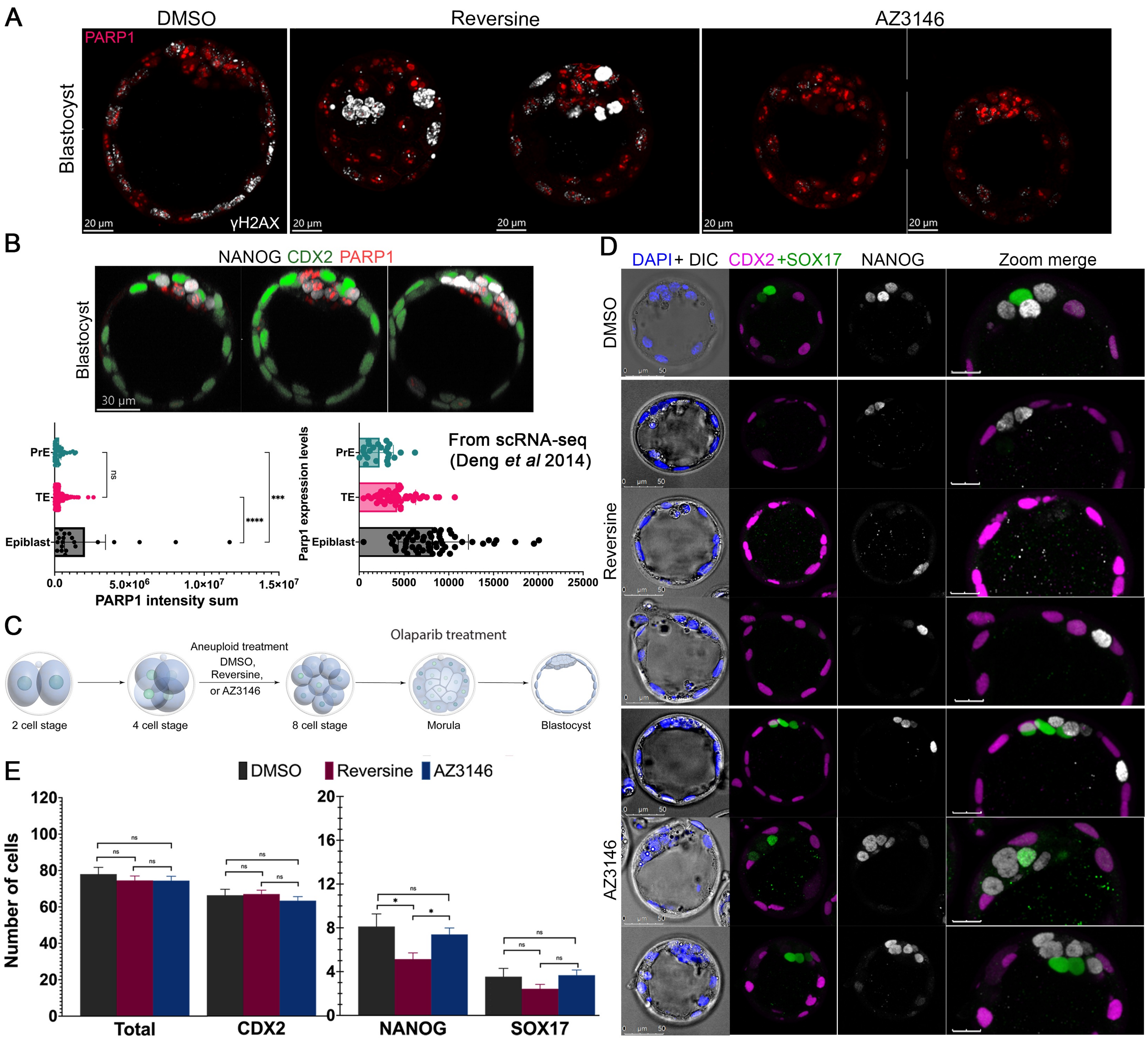

Figure S. 3

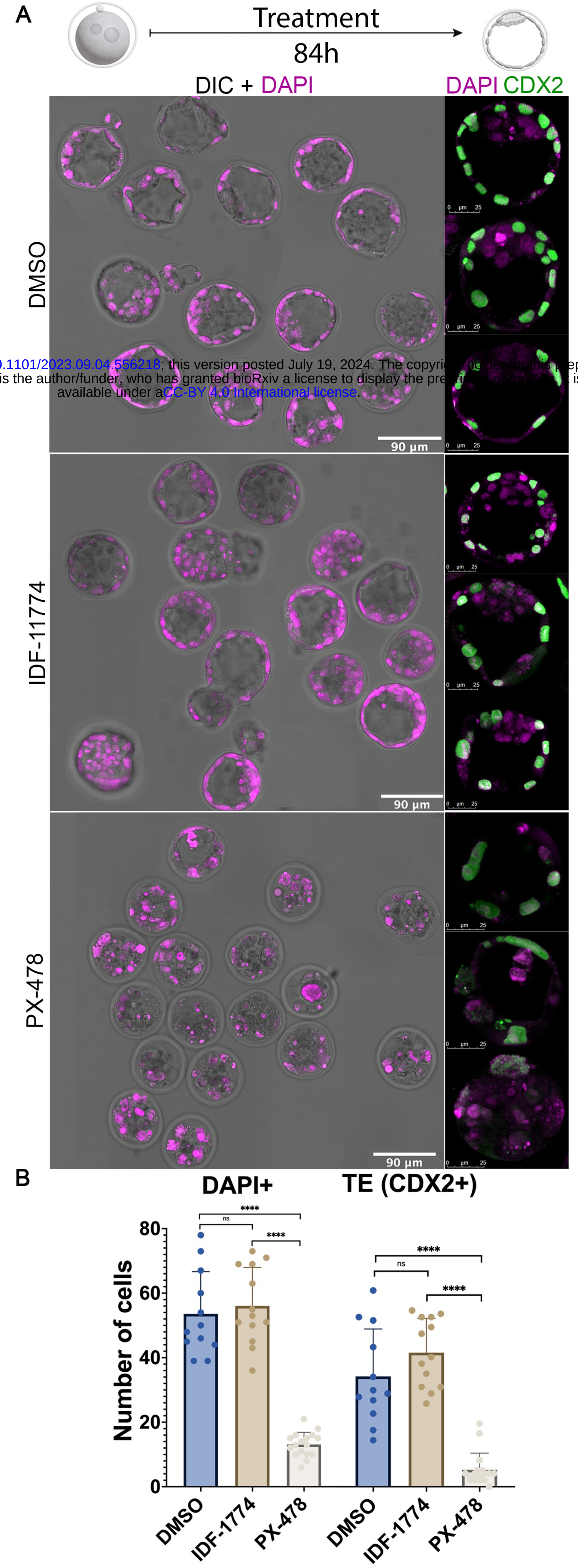

bioRxiv preprint doi: https://doi.org/10.1101/2023.09.04.556218; this version posted July 19, 2024. The copyright holder for this preprint (which was not certified by peer review) is the author/funder, who has granted bioRxiv a license to display the preprint in perpetuity. It is made available under aCC-BY 4.0 International license.

Figure S. 4

A

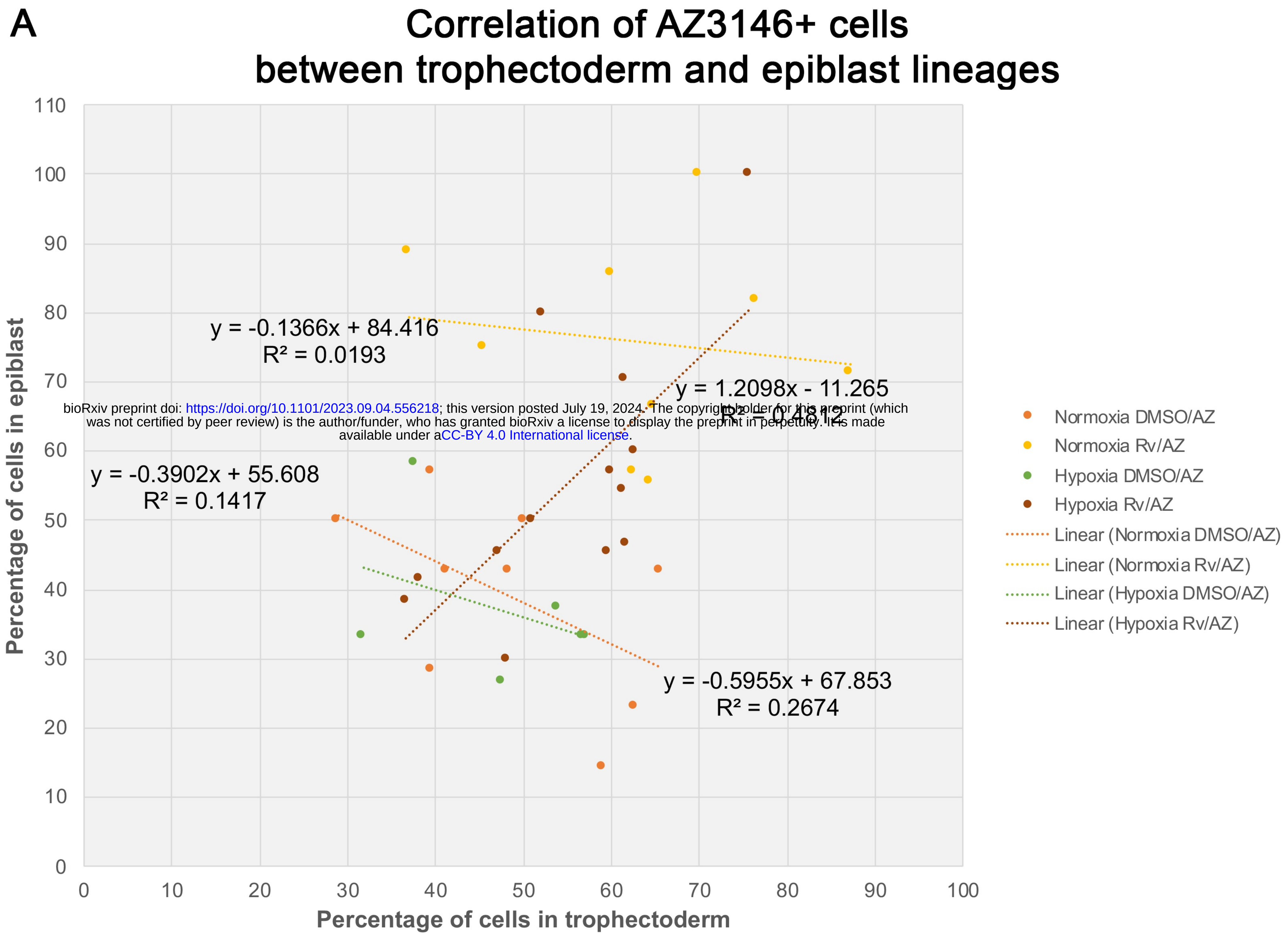

bioRxiv preprint doi: https://doi.org/10.1101/2023.09.04.556218; this version posted July 19, 2024. The copyright holder for this preprint (which was not certified by peer review) is the author/funder, who has granted bioRxiv a license to display the preprint in perpetuity. It is made available under aCC-BY 4.0 International license.

Figure S. 5

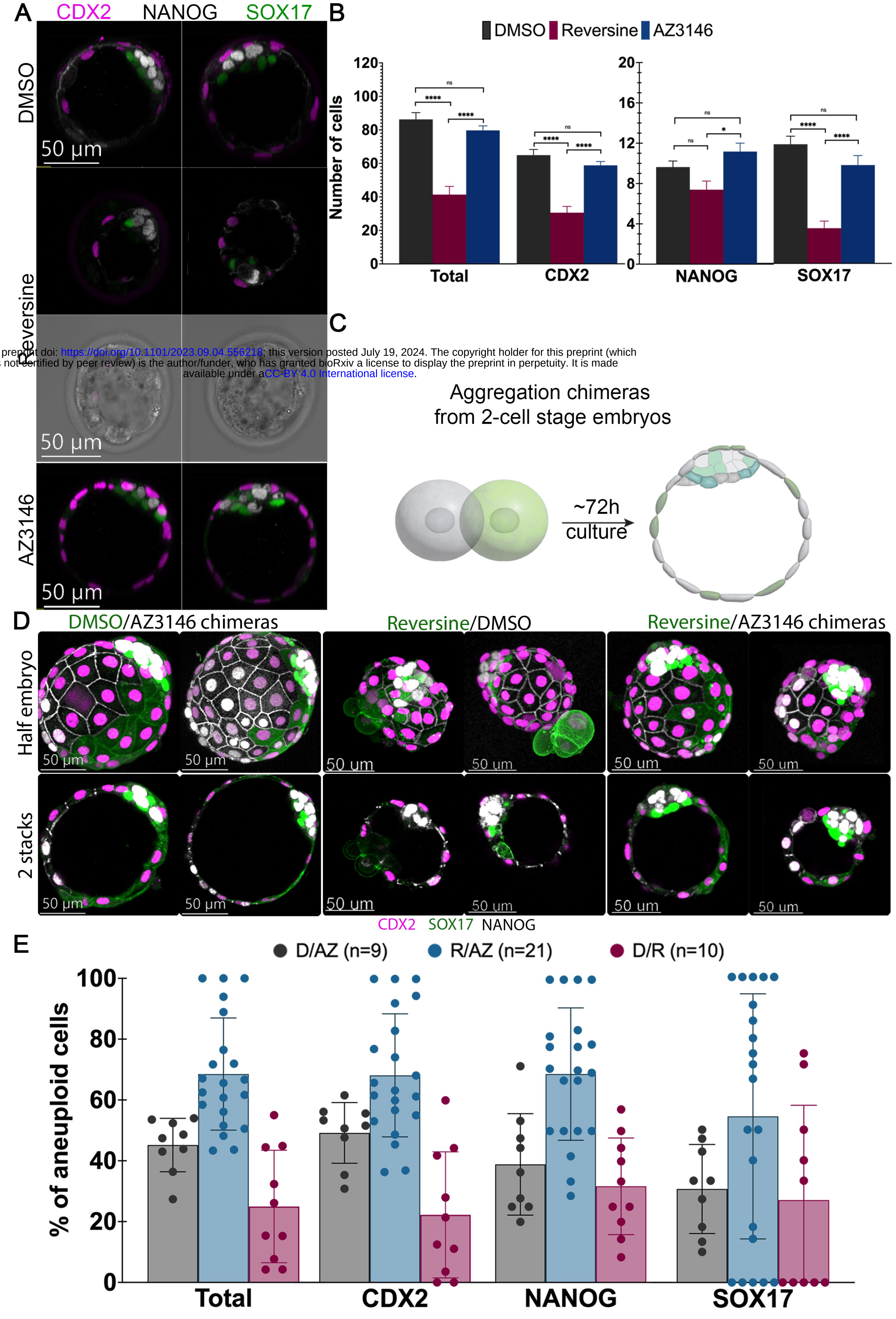

bioRxiv preprint doi: https://doi.org/10.1101/2023.09.04.556218; this version posted July 19, 2024. The copyright holder for this preprint (which was not certified by peer review) is the author/funder, who has granted bioRxiv a license to display the preprint in perpetuity. It is made available under aCC-BY 4.0 International license.

bioRxiv preprint doi: https://doi.org/10.1101/2023.09.04.556218; this version posted July 19, 2024. The copyright holder for this preprint (which was not certified by peer review) is the author/funder, who has granted bioRxiv a license to display the preprint in perpetuity. It is made available under aCC-BY 4.0 International license.

Figure S. 6

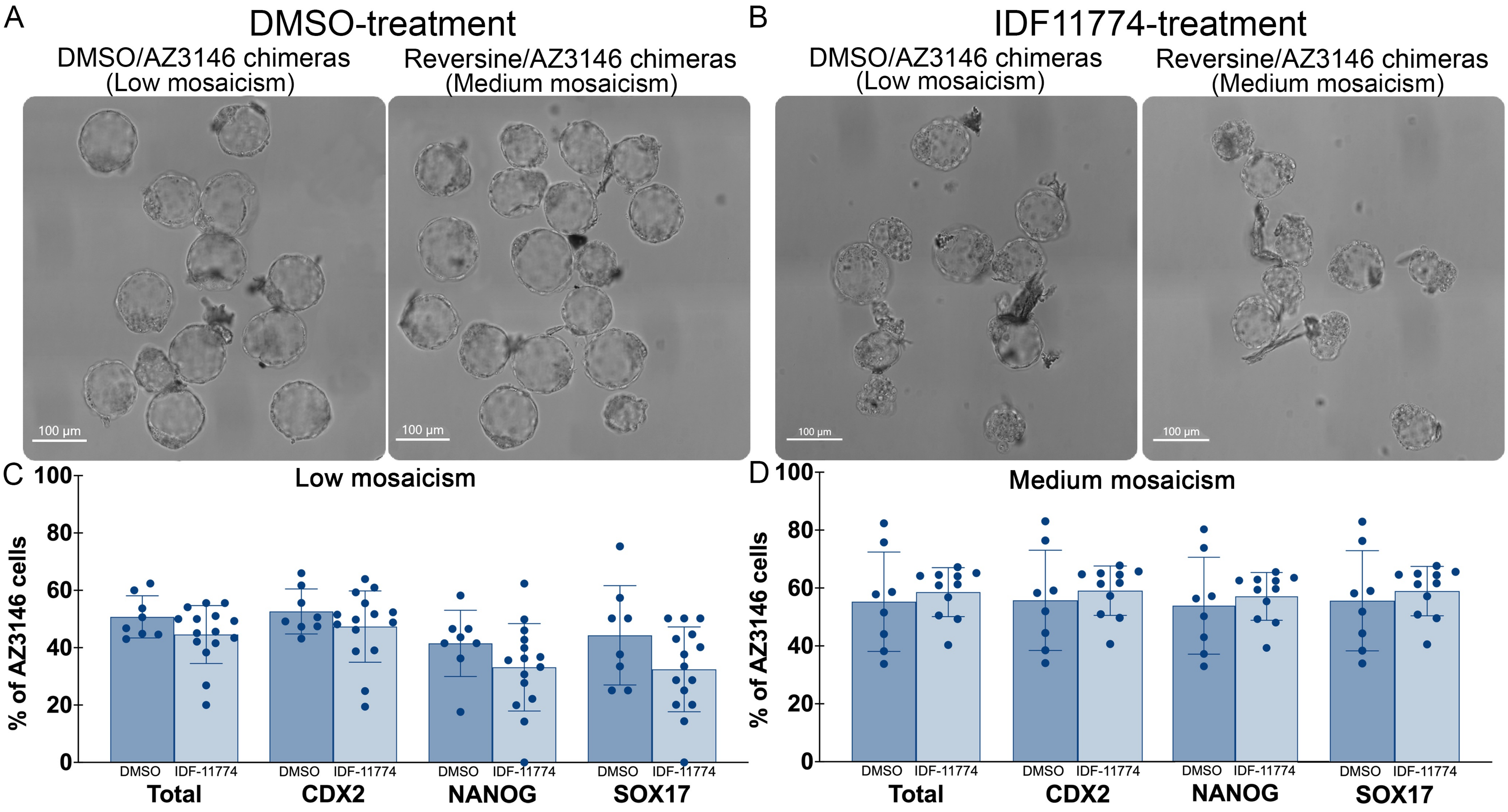

Supplement: Supplement 1 [file NIHPP2023.09.04.556218v2-supplement-1.pdf]
